# Conductivity Rise During Irreversible Electroporation: True Permeabilization or Heat?

**DOI:** 10.1007/s00270-018-1971-7

**Published:** 2018-04-23

**Authors:** Alette H. Ruarus, Laurien G. P. H. Vroomen, Robbert S. Puijk, Hester J. Scheffer, Theo J. C. Faes, Martijn R. Meijerink

**Affiliations:** Department of Radiology and Nuclear Medicine, VU University Medical Center, De Boelelaan 1117, 1081 HV Amsterdam, The Netherlands

**Keywords:** Irreversible electroporation, Conductivity, Permeabilization, IRE, Temperature

## Abstract

**Purpose:**

Irreversible electroporation (IRE) induces apoptosis with high-voltage electric pulses. Although the working mechanism is non-thermal, development of secondary Joule heating occurs. This study investigated whether the observed conductivity rise during IRE is caused by increased cellular permeabilization or heat development.

**Methods:**

IRE was performed in a gelatin tissue phantom, in potato tubers, and in 30 patients with unresectable colorectal liver metastases (CRLM). Continuous versus sequential pulsing protocols (10-90 vs. 10-30-30-30) were assessed. Temperature was measured using fiber-optic probes. After temperature had returned to baseline, 100 additional pulses were delivered. The primary technique efficacy of the treated CRLM was compared to the periprocedural current rise. Seven patients received ten additional pulses after a 10-min cool-down period.

**Results:**

Temperature and current rise was higher for the continuous pulsing protocol (medians, gel: 13.05 vs. 9.55 °C and 9 amperes (A) vs. 7A; potato: 12.70 vs. 10.53 °C and 6.0A vs. 6.5A). After cooling-down, current returned to baseline in the gel phantom and near baseline values (Δ2A with continuous- and Δ5A with sequential pulsing) in the potato tubers. The current declined after cooling-down in all seven patients with CRLM, although baseline values were not reached. There was a positive correlation between current rise and primary technique efficacy (*p* = 0.02); however, the previously reported current increase threshold of 12–15A was reached in 13%.

**Conclusion:**

The observed conductivity rise during IRE is caused by both cellular permeabilization and heat development. Although a correlation between current rise and efficacy exists, the current increase threshold seems unfeasible for CRLM.

**Electronic supplementary material:**

The online version of this article (10.1007/s00270-018-1971-7) contains supplementary material, which is available to authorized users.

## Introduction

Electroporation is a phenomenon that occurs when strong electric field pulses are applied to cells, thereby altering the transmembrane potential, and eventually leading to the formation of nanopores in the cellular membrane [1–3]. Depending on the magnitude of the electrical field pulses, frequency and exposure time to the electrical field, the permeabilization of the cellular membrane can be temporary (reversible electroporation) or permanent (irreversible electroporation; IRE) [4]. Reversible electroporation has been used to introduce genes and drugs into the cell, after which the cellular membrane recovers [5, 6]. In IRE, the combination of a high-magnitude electrical field and long exposure time to this electrical field is used, leading to permanent disrupted cell membranes and eventually apoptosis [7].

IRE has been investigated as treatment option for a variety of malignant tumors, and safety has been confirmed in several studies [8–12]. Although IRE is currently used as a ‘last resort treatment’ (patients would otherwise be designated to palliative treatment), efficacy results are moderate [10, 13–15]. Local tumor progression (LTP) after IRE ranges between 55 and 93% for colorectal liver metastases (CRLMs) [11]. Therefore, it is desirable to obtain prognostic factors indicating complete ablation during the procedure to improve oncologic outcome. However, in contrast to thermal-based ablation techniques such as radiofrequency and microwave ablation, temperature development and exposure time to temperature are not feasible as endpoints of successful ablation with IRE [16]. Furthermore, imaging directly after the procedure is not reliable due to edema, artifactual distortion, and formation of gas pockets during IRE [17].

**Fig. 1:**
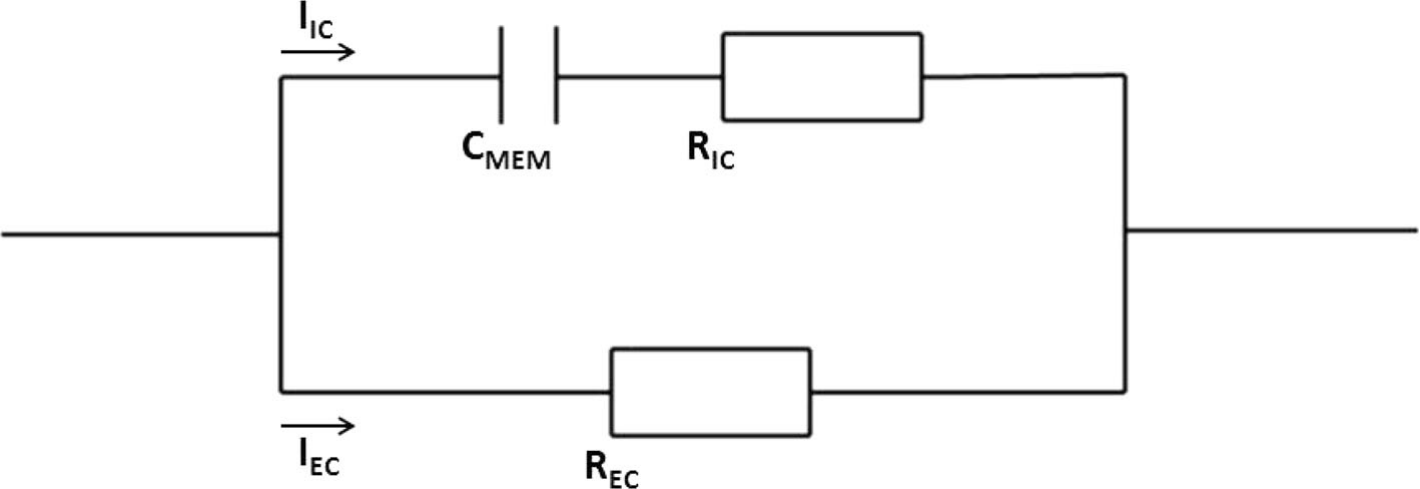
Simple electric circuit model of biological tissue. I = current; IC = intracellular; EC = extracellular; C_MEM_ = capacitance cell membrane; R = resistor

Several studies have investigated methods to predict real-time treatment effectiveness [17–19]. An observed current rise during IRE of 12–15 amperes (A) has been suggested as indicator for successful ablation [17, 18]. This proposed method is based on the assumption that electrical properties of ablated tissue are altered by disruption of the cellular membranes in an ablation zone [17, 18]. By using an electric circuit model of tissue, the effects of electroporation on the electrical properties of ablated tissue can be illustrated (Fig. 1). Prior to electroporation, the current passes through the extracellular space, since the membrane capacitance prevents it from passing intracellularly. After electroporation, the induced nanopores shunt the membrane capacitance, resulting in a lower cell resistance, because both the parallel connected intracellular and extracellular resistances (*R*_IC_ and *R*_EC_) contribute to the current conduction. Given this fact, the periprocedural current rise as an endpoint for IRE success is explained using Ohm’s law ($I = \frac{U}{R}$), as the voltage is set and the resistance decreases.

However, the conductivity of human tissue is also affected by temperature. Although IRE was initially considered non-thermal, recent papers have shown a certain temperature rise during IRE due to secondary Joule heating [4, 20–22]. The correlation between tissue temperature and electrical conductivity can be characterized by the following Eq. (1):

1 $$\sigma \left( T \right) = \sigma_{0} \left[ {1 + \kappa_{1} \left( {T - T_{0} } \right)} \right]$$

in which *σ*(*T*) is the conductivity (Siemens/meter) at temperature *T*(°C), *σ*_0_ is the equivalent conductivity at ambient temperature *T*_0_, and *κ*_1_ is the temperature coefficient of tissue, typically in the order of 2%/°C for aqueous solutions [23, 24]. Hence, electrical conductivity increases from *σ*_0_ to σ for a temperature increase of *T*_0_ to *T*. This conductance increase is physically explained by the fact that fluid viscosity decreases with increasing temperature, meaning increased mobility of the involved ionic charge carriers. Summarizing, when temperature increases, electric tissue resistance decreases and electric current increases.

Based on both formulas, our hypothesis and primary study objective was that the periprocedural conductivity rise may not be solely attributable to the decrease in resistance caused by disrupted cell membranes, but also to a tissue temperature increase secondary to Joule heating. Our secondary objective was to investigate whether current increase and oncologic outcome are correlated in IRE for CRLM.

## Materials and Methods

### In Vitro Experiments

**Fig. 2:**
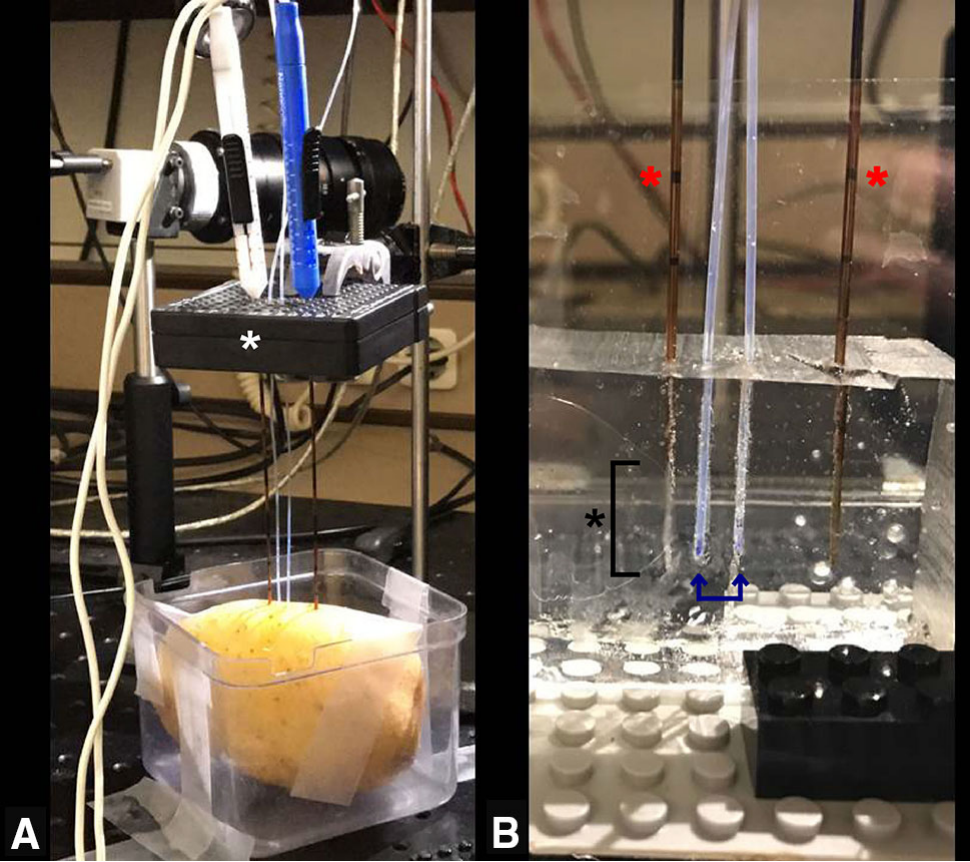
Setup of in vitro IRE experiments. **A** Two needle–electrodes were inserted parallel in the potato through an external spacer (white asterisk), ensuring inter-electrode distance of 2 cm and parallelism; **B** close-up image of the needle–electrodes (red asterisks) inserted in the gel phantom with two fiber-optic temperature probes (blue arrows) inserted at 0.5 and 1.0 cm distance from the electrode tip (black asterisk at bracket: active tip of the electrode)

An acellular gelatin tissue phantom and a potato tuber model were used (Fig. 2). The tissue phantom was made of 150 ml saline (NaCl 0.9%), 125 mg ammonium persulfate, 100 ml 30% acrylamide/bis solution and 200 μl tetramethylethylenediamine, and mimicked soft biological tissue with regard to its electrical properties [25]. Potato tubers are an established medium for research in electroporation, since any irreversibly electroporated area will be distinctively darker hours after electroporation due to the release of an enzyme called polyphenoloxidase through damaged cell membranes [26].

#### Treatment Protocol

Electrodes were placed parallel to each other with an inter-electrode distance of 2.0 cm, active tip length of 2.0, and 0.5 cm distance to the gel surface. To investigate whether the type of pulse protocol affects current increase, ninety pulses were either delivered continuously or sequentially in 3 cycles of 30 pulses, with a pulse length of 90 μ sec, and a voltage-to-distance ratio of 1000 V/cm. To measure the temperature within the ablated area, two fiber-optic temperature probes, with a 1 mm diameter (TRUE Lumiterm X5, Ipitek, Carlsbad, CA, USA), were placed 5 mm from an electrode tip and right in the middle of the two electrodes (Fig. 2). Temperature was measured prior to, during and after pulse delivery until it returned to baseline. Since baseline temperature of the gel phantoms and potato tubers varied, the temperature change (Δ*T*) was determined between baseline and maximum temperature measured. After a sufficient cool-down period, in which the temperature returned to baseline, the same pulse protocol was repeated. Furthermore, the sequential pulse protocol was applied in a preheated gel phantom (comparing 20 and 40 °C). Additional data on hypotheses are given in Online Resource 1. All data regarding delivered current and voltage settings were obtained from the NanoKnife system.

### In Vivo Experiments

An evaluation of patients treated with percutaneous IRE for CRLM was performed. The cohort consisted of participants from the prospective COLDFIRE-II trial (ClinicalTrials.gov number NCT02082782) [27]. The trial was approved by the Medical Ethical Review Board of the VU University Medical Center. Inclusion criteria were small (≤ 3.5 cm) ^18^F-fluorodeoxyglucose positron emission tomography–computed tomography (^18^F-FDG PET–CT) avid CRLM, unsuitable for resection or thermal ablation due to proximity to vital structures. The procedure was performed using the NanoKnife system^®^ (AngioDynamics, Latham, New York) and has previously been described [28].

#### Treatment Protocol

The same pulse protocol was used as for the in vitro protocol. Patients treated with sequential pulsing (10-30-30-30 pulses) were compared with patients treated with continuous pulsing (10-90), which had been adjusted/incorporated during a routine protocol alteration in the COLDFIRE-II trial.

In seven patients, the standard pulses were followed by a 10-min cool-down period, after which another 10 pulses were admitted. The resistance after this 10-min pause was calculated from the maximum current that arose during the last cluster pulses and the set voltage. This value was compared to the resistance at the beginning and end of the standard procedure.

#### Correlation Between Efficacy and Current Increase

**Fig. 3:**
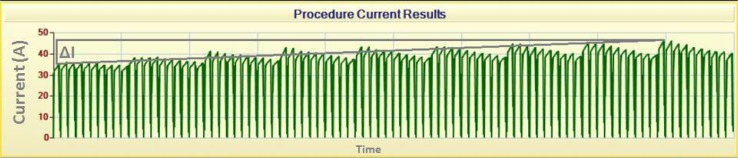
Current increase during IRE. Image obtained from the NanoKnife system showing (1) a current decrease within a cluster of pulses; (2) a current increase (Δ*I*) of around 11 A between the first and last cluster

The increase in current (Δ*I*) was calculated for each needle–electrode pair by subtracting the maximum current of the first cluster pulses (i.e., 10 pulses) from the maximum current of the last cluster (Fig. 3). In case of over- or undercurrent, the voltage settings were adjusted manually. To compare Δ*I* at the beginning and end of the procedure between individual patients, resistance was calculated using Ohm’s law dividing the voltage by the maximum current. Next, the difference in resistance between the first and last clusters was calculated (Eq. 2), and the mean decrease in resistance was determined for all needle–electrode pairs together (Eq. 3).

2 $$\Delta R_{\text{pair}} = R_{\text{end}} - R_{\text{begin}}$$

3 $$\Delta R_{\text{total}} = \left( {\Delta R_{{{\text{pair}} 1}} + \Delta R_{{{\text{pair }}2}} + \cdots + \Delta R_{{{\text{pair}} n}} } \right) \div n$$

Subsequently, the mean decrease in resistance was converted to the corresponding difference in current at a standardized voltage of 3000 V (Eq. 4), to compare subjects.

4 $$\Delta I_{{3000{\text{V}}}} = \frac{3000}{{\Delta R_{\text{total}} }}$$

Patients with and without LTP were compared to investigate a correlation between efficacy of IRE and the current increase. If patients had at least a ^18^F-FDG PET-CT scan at 3 months, they were included in the analysis [29].

### Statistical Analysis

Continuous variables were presented with standard descriptive statistics including means, standard deviations, medians and ranges. Categorical variables were presented with frequencies. A Pearson’s correlation was computed to assess the relationship between current and temperature increase. Student’s *t* test for independent samples was used to compare current increase in patients with and without LTP. A *p* value < 0.05 was considered statistically significant. Data were analyzed using SPSS version 22.0.

## Results

### In Vitro Experiments

**Fig. 4:**
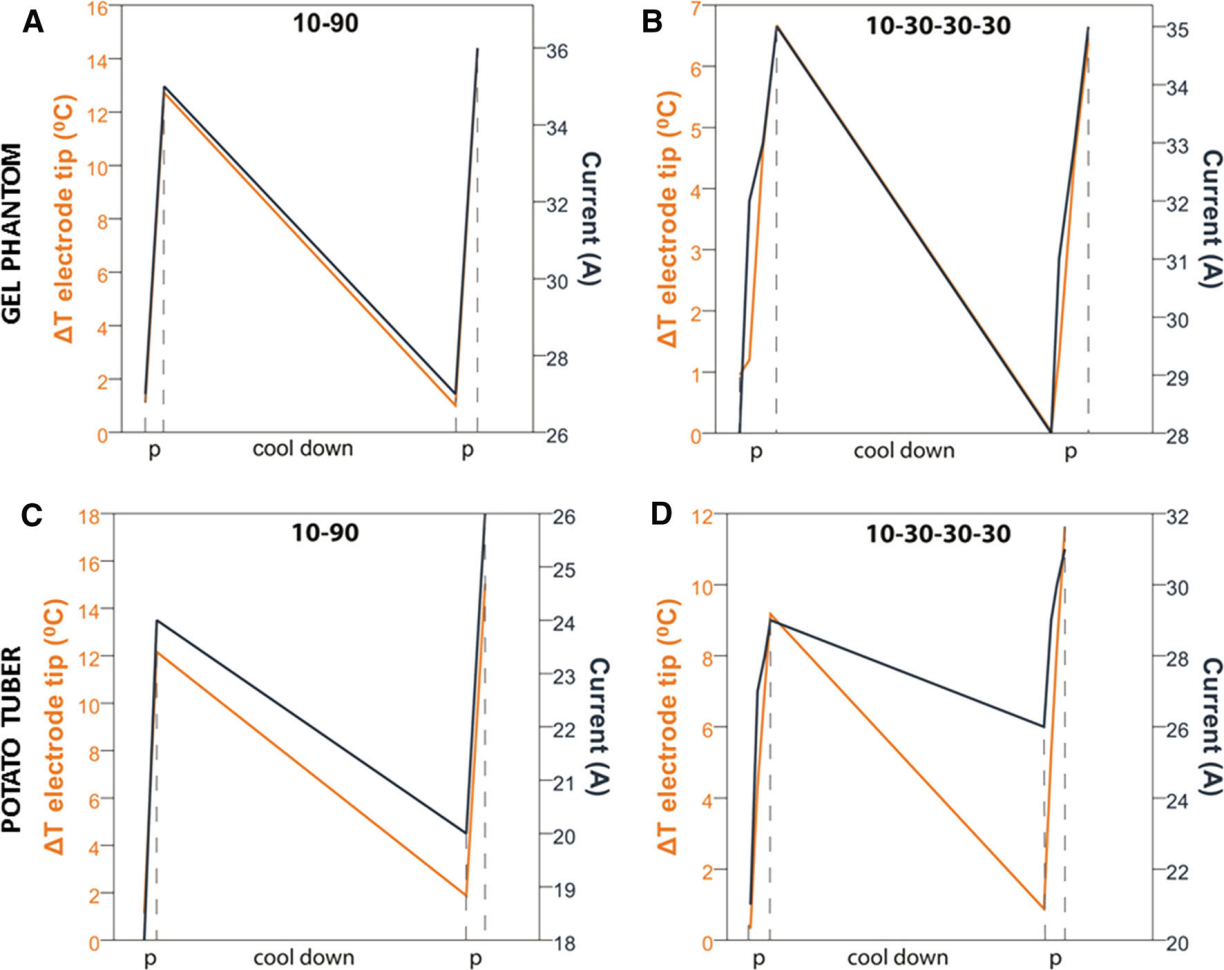
Temperature (orange line) and current (dark blue line) development during in vitro IRE. **A**, **B** Experiments in gel phantom including cool-down; **C, D** Experiments in potato tuber including cool-down. *Note* the differences in scales. The data are linearly interpolated between the data points

**Fig. 5:**
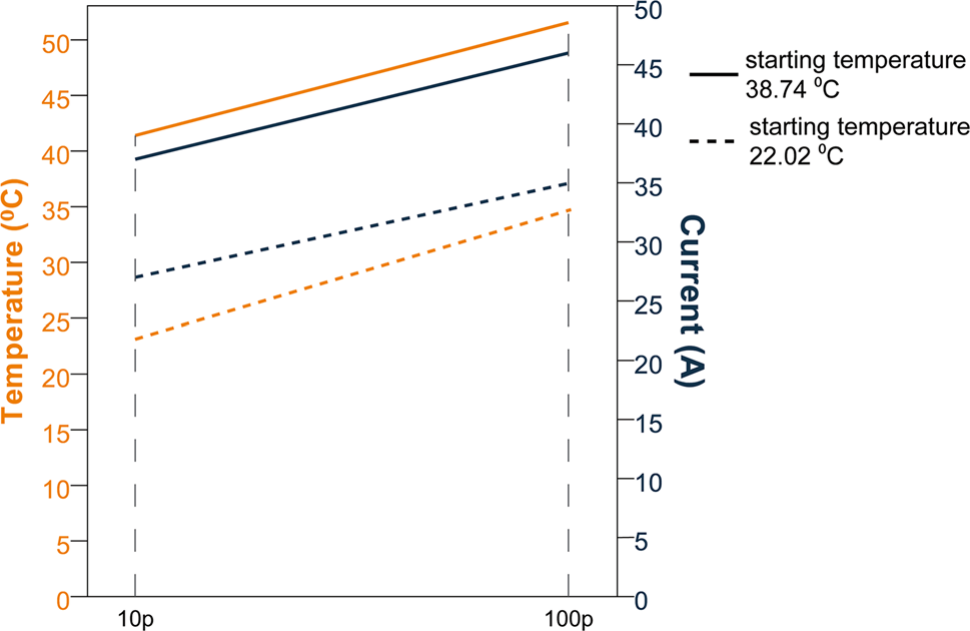
Temperature (orange line) and current (dark blue line) development during in vitro IRE with different starting temperatures of the gel phantom. Solid line: starting temperature 38.74 °C; dashed line: starting temperature 22.02 °C. *Note* the graphs are linearly interpolated between 10 and 100 pulses

During all in vitro experiments, a temperature increase was recorded, which was higher at 5 mm from the electrode tips compared to the area in the middle of the electrode tips (10 mm). Median Δ*T* was higher during continuous pulsing (*p* = 0.007 at 5 mm and *p* = 0.018 at 10 mm) than during sequential pulsing, although the latter was not statistically significant (*p* = 0.074 at 5 mm and *p* = 0.894 at 10 mm). Medians of maximum Δ*T* are shown in Table 1 (note the small ranges compared to the difference of the medians). Median Δ*I* per pulse protocol is given in Table 2. Median cooling-down time was 37 min. After this cool-down period, amperage decreased to baseline value in the gel phantom. In the potato tubers, amperage declined but did not reach baseline value (Δ2A with continuous- and Δ5A with sequential pulsing). Δ*I* was positively correlated with temperature increase for both mediums and pulse protocols (gel phantom *p* < 0.001 (continuous) and *p* = 0.002 (sequential); potato tuber *p* = 0.019 (continuous) and *p* = 0.005 (sequential); Fig. 4). When the baseline temperature of the gel phantom was preheated to 40 °C, the starting amperage was higher compared to a baseline temperature of 20 °C (Fig. 5).

**Table 1:** Temperature increase during in vitro IRE

	Continuous pulsing (10-90 pulses)	Sequential pulsing (10-30-30-30 pulses)	*p* value
Median (range) of the maximum temperature increase in °C			
Electrode tip (5 mm)			
Gel phantom (20 °C)	13.05 (12.72–13.29)	9.55 (9.52–9.57)	0.007
Potato tubers	12.70 (12.15–13.25)	10.53 (10.22–10.83)	0.074
Between electrodes			
Gel phantom (20 °C)	5.86 (5.75–5.96)	4.99 (4.93–5.04)	0.018
Potato tubers	7.91 (7.74–8.07)	7.88 (7.86–7.90)	0.894

**Table 2:** Current increase during in vitro IRE

	Continuous pulsing (10-90 pulses)	Sequential pulsing (10-30-30-30 pulses)
Median (range) increase in current in amperes		
Gel phantom (20 °C)	9.0 (9.0–9.0)	7.0 (7.0–7.0)
Potato tubers	6.0 (6.0–6.0)	6.5 (5.0–8.0)

### In Vivo Experiments

**Fig. 6:**
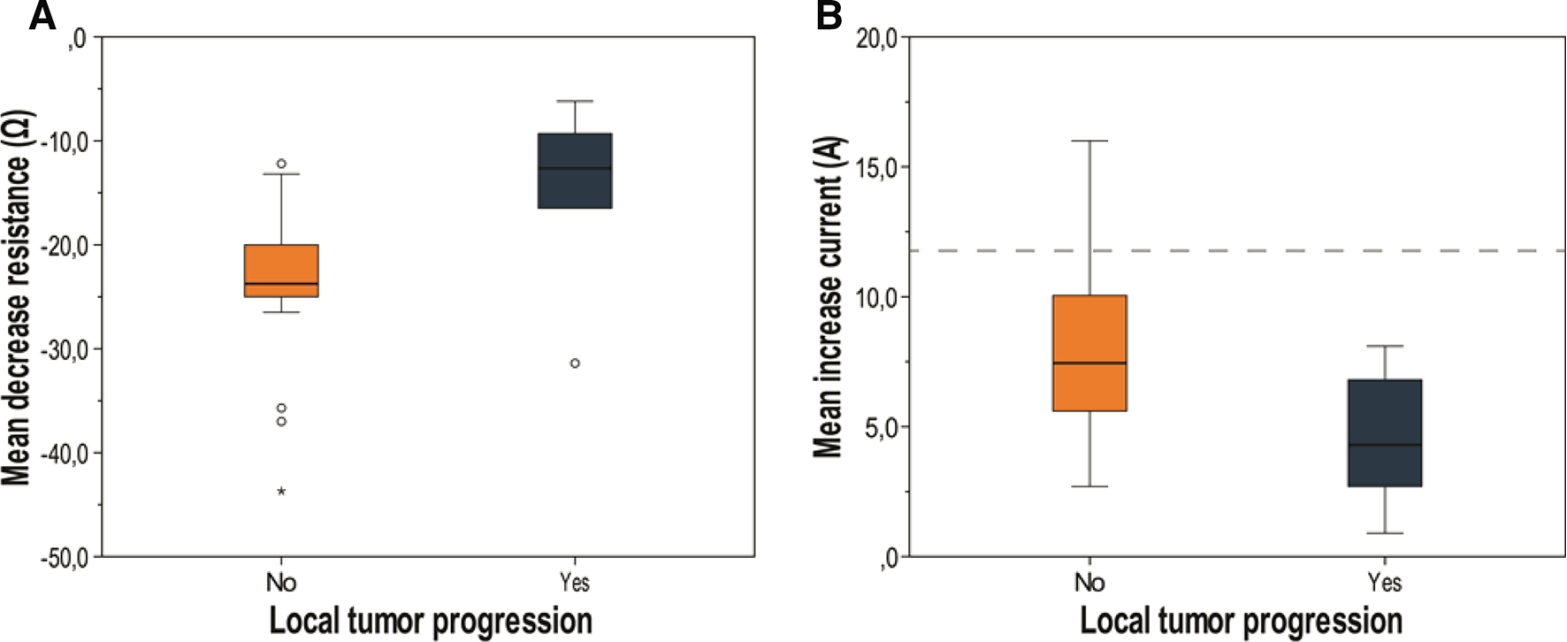
Box plots showing mean decrease in periprocedural resistance (**A**) and mean periprocedural increase in current (**B**) of patients with (green box) and without LTP (orange box). The dashed line is set at 12A

Thirty patients were evaluated. Baseline characteristics are summarized in Table 3. After a median follow-up period of 11.5 months (range 3–30 months), six patients showed LTP (20%). LTP occurred relatively late after treatment, ranging from 5 to 23 months. All patients showed a decrease in resistance during IRE (mean 21.6 Ω; range 6.2–43.7 Ω) and subsequently an increase in current (mean 7.3A; range 0.9–16.0A). Patients with LTP showed a significantly lower decrease in resistance (14.8 Ω) compared to patients without LTP (23.3 Ω; *p* = 0.02; Fig. 6A). This finding was correlated with a significantly higher current increase in patients without LTP (8.0 vs. 4.5A; *p* = 0.02; Fig. 6B). The mean current increase during continuous pulsing was lower than during sequential pulsing (6.2 vs. 8.2A); however, this finding was not statistically significant (*p* = 0.09).

**Table 3:** Characteristics of all patients treated with IRE for CRLM

	LTP (*n* = 6; 20%)	No LTP (*n* = 24; 80%)	*p* value
Gender			
Male	3	18	0.25
Female	3	6	
Size longest diameter; mm, median (range)	19.0 (10.0–35.0)	21.5 (10.0–38.0)	0.94
# of electrodes, median (range)	4 (3–7)	4 (2–7)	0.54
Pulse protocol			
Continuous	2	11	0.60
Sequential	4	13	
Approach			
Open	0	3	0.48
Percutaneous	6	21	
Current ampere, mean (range)			
Begin	31.1 (23.7–38.8)	25.2 (18.0–33.0)	
End	35.6 (30.3–41.5)	33.3 (24.6–42.7)	
Increase	4.5 (0.9–8.1)	8.0 (2.7–16.0)	0.02
Resistance ohm, mean (range)			
Begin	91.4 (77.4–125.6)	103.0 (72.8–133.0)	
End	76.7 (65.2–94.1)	79.6 (60.6–99.3)	
Decrease	14.8 (6.2–31.4)	23.3 (12.2–43.7)	0.02

**Fig. 7:**
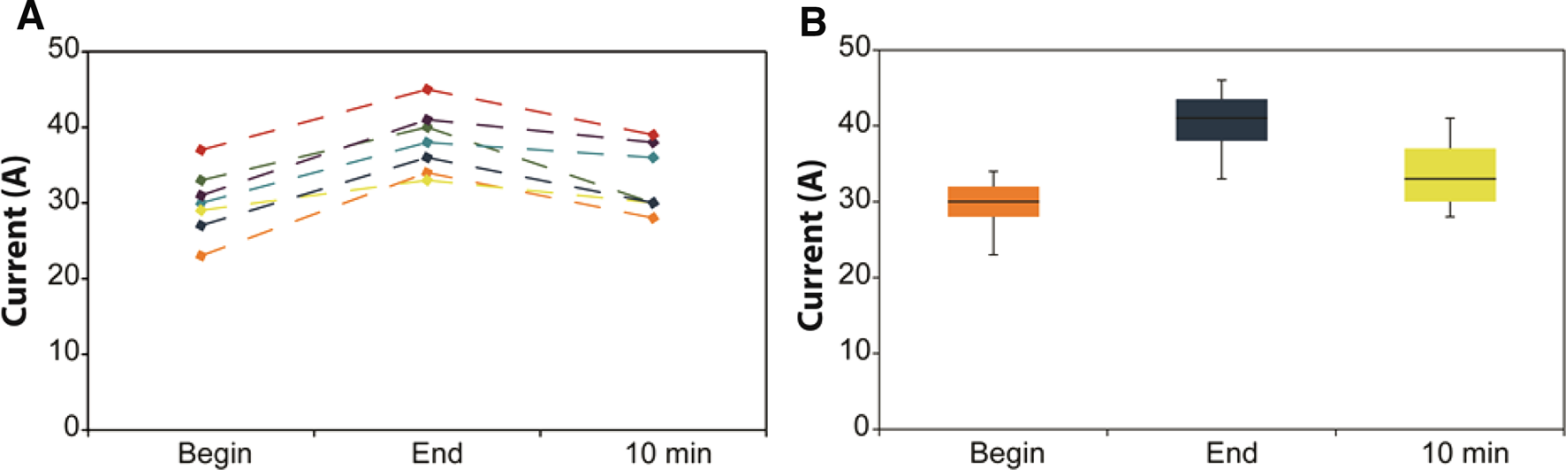
Current development in patients. **A** data per individual patient; **B** box plot of current development of all patients; begin: measured current after the first 10 pulses, end: measured current after 100 pulses, 10 min: measured current after the 10 pulses following a 10-min pause

Seven patients received 10 additional pulses after a 10-min cool-down period. All patients showed a current increase during the standard pulse protocol. The current after the 10-min pause was lower compared to the current after 90 pulses (Fig. 7).

## Discussion

This study was performed to examine whether the observed current rise during treatment with IRE is based upon cell permeabilization within the ablation zone and/or upon temperature rise-related tissue conductivity increase. Both in the tissue-mimicking gel phantom and in the potato tuber, we observed a rise in current and temperature. These findings suggest that the current increase cannot solely be held accountable to membrane permeabilization, because in an acellular phantom permeabilization is absent. Furthermore, when temperature returned to baseline after the cool-down phase, the amperage of the following delivered pulses also returned to baseline values in the tissue-mimicking gel phantom and declined in the potato tubers. This suggests that temperature increase that occurs during IRE plays a part in the current increase observed with IRE. The association between higher starting amperage and higher baseline temperature of the gel phantom also supports the hypothesis that current increase depends on temperature (Fig. 5).

After evaluation of 30 patients treated with IRE for CRLM, patients with LTP showed a statistically significant lower decrease in resistance and subsequently a low increase in current during IRE compared to patients without LTP. This is in accordance with the study performed by Dunki-Jacobs et al., who found a correlation between amperes increase and local recurrence after pancreatic IRE. They suggested a minimum current increase of 12–15A for successful ablation, with repetition of the protocol in case of a lower increase [17]. However, in the subset of patients without LTP in our study, only 3 patients (10%) showed an increase of at least 12A. Repeating the protocol in the remaining 21 patients without LTP (70%), as suggested by Dunki-Jacobs et al., would have implicated overtreatment and an increased risk of thermal damage caused by the accumulated energy of the additional pulses [1, 30]. A possible explanation for the low number of patients who experienced 12A increase during IRE, could be the different tumor histology (CRLM vs. pancreatic cancer) [17]. These conflicting results suggest that the implementation of this value as feasible endpoint for successful ablation might be premature to apply to all tumor types [31]. Furthermore, it has never been established that applying additional pulses after 100 pulses will increase the ablation zone [32]. In fact, one could even hypothesize that the additional current increase during the extra pulses is ascribed to higher temperatures, rapidly increasing the risk for thermal damage.

If the conductivity increase depends on temperature increase, it is expected that the current increase in patients treated with continuous pulsing would be higher compared to sequential pulsing. However, there was no significant difference found in current increase between both pulse protocols. This finding could be explained by the observation that the median time for cooling down in the in vitro experiments was 37 min. So, the short pause of several seconds until approximately 1 min between the consecutive 30 pulses during sequential pulsing in clinical patients may be too short to establish a temperature decrease to baseline values. However, living tissue is perfused and will conduct temperatures faster and further away from the ablation zone compared to the gel phantom, shortening the cool-down period in vivo [33].

Another explanation could be the greater electroporation effect with sequential pulsing. Appelbaum et al. [34] showed that sequential pulsing with a four-probe array created larger ablation zones than continuous pulsing. The authors hypothesized that the increase in conductivity induced by an IRE pulse persists after the initial pulse. The shifts of cellular contents, caused by the opening of IRE-induced nanopores in the cellular membrane, occur in the order of minutes rather than seconds. So, timing of pulses might also influence ablation zone volume, due to increased electroporation effects with sequential pulsing. The greater electroporation effect will increase conductivity, but this effect is compensated by the lower increase in temperature compared to continuous pulsing, which causes a smaller electroporation effect but greater temperature increase.

After the 10-min pause in patients, the current decreased in all patients but did not reach baseline values. If the conductivity rise would be explained by permeabilization of cell membranes, the pause would not have caused any decrease in current in the following pulses, since the permeabilization would be irreversible. However, if the conductivity only depends on temperature, the current would return to baseline values. This finding suggests that both permeabilization *and* temperature affect the current increase during IRE.

There is a potential flaw in the assumption that occurrence of LTP could entirely be prevented by reaching an intraprocedural endpoint indicating successful ablation, while needle placement also plays an essential part in the occurrence of LTP. IRE typically creates a sharply demarcated ablation zone, killing all cells within, making it well suited for destroying the entire visible tumor [35]. However, at the time of treatment there may already be infiltrative cells outside the borders of the visible tumor. This fact also contributes to the occurrence of LTP, since pre-treatment planning of the needle-electrodes is based on visible tumor. The ablate-and-resect study performed by Scheffer et al. showed the existence of a reversibly electroporated tissue zone between the irreversibly damaged ablation zone and the normal liver parenchyma. Combining IRE with systemic or electrochemotherapy could eradicate the infiltrative cells just outside the margin of the ablation zone, thereby decreasing the occurrence of LTP [36, 37].

This study has its own limitations. The temperature distribution in the gel phantom and potato tubers will probably differ from in vivo temperature distribution, since both in vitro mediums were colder than normal tissue and unperfused. Nevertheless, temperature development was consistently positively correlated with current development. Furthermore, a methodical drawback of the study is the short follow-up period (3 months) for some patients. Although the median follow-up of 11.5 months is potentially long enough to properly categorize patients in the subgroups with or without LTP, it could be possible that some patients developed LTP after their last follow-up and are therefore falsely counted as LTP free. Another important limitation of the current study is that the mean current rise of all needle–electrode pairs together was calculated, possibly leading to under- or overestimation of this value by compensating for a needle–electrode pair that shows little or high rise in current. Therefore, we are currently remodeling the needle placement in all treated patients, to investigate whether LTP can be predicted by a low increase in current between one needle–electrode pair. Furthermore, we will retrospectively visualize the electric field distribution based upon needle placement and applied electric parameters, to investigate whether this would have predicted LTP and could be used to prevent LTP prospectively.

This study did not take electrolysis into account as phenomenon occurring during electroporation. Previous studies have shown that with increasing number of pulses, electrolytically produced gases shape along the electrodes [22, 38, 39]. Guenther et al. [38] suggested that the sudden increase in current at the start of a cluster of pulses is caused by a violent discharge when the electric field across the gas layers increases above the breakdown voltage. The currently used clinical protocols for IRE, i.e., a large number of high-voltage pulses, cannot distinguish which phenomenon (temperature development or electric breakdown) causes the increase in current. Suggestions have been made to avoid the electric breakdown and subsequent surges in current, by delivering the pulses as continuously decreasing voltages to avoid that the voltage is sufficient to produce the breakdown or by delivering the pulses at a lower frequency to facilitate diffusion of the gases. However, both suggestions will also decrease temperature development with IRE, thus not allowing distinction between the causes of current increase [22]. Though the relevant question would be whether the pulse protocol can be adjusted in a way that tissue is still irreversibly damaged and efficacy results remain the same, no electric breakdown occurs, since associated pressure waves can cause substantial damage to surrounding healthy tissue, and temperature increase is limited to a minimum.

In conclusion, this study has shown that the observed conductivity rise during IRE is not solely attributable to electroporation of cell membranes, but also subsidiary to development of heat. As previously mentioned, a significant difference was found in resistance decrease and subsequently current increase in patients with and without LTP [17]. However, the widespread adoption of this value may have been premature, since the suggested required current increase of 12–15A would have implicated overtreatment of a large subset of patients, unnecessarily increasing the risk of thermal damage. To ensure complete ablation while preventing thermal damage, the search toward optimized clinical pulse protocols for all various tumor types should still continue.

## Electronic Supplementary Material

Below is the link to the electronic supplementary material.

Supplementary material 1 (DOCX 15 kb)

## Abbreviations

^18^F-FDG PET–CT: ^18^F-fluorodeoxyglucose positron emission tomography–computed tomography
A: Amperes
CRLM: Colorectal liver metastasis
IRE: Irreversible electroporation
LTP: Local tumor progression
