# Supplementary material for: Conductivity Rise During Irreversible Electroporation: True Permeabilization or Heat?
Source: Cardiovasc Intervent Radiol. 2018 Apr 23;41(8):1257–66. doi: 10.1007/s00270-018-1971-7 (PMC6021471; doi:10.1007/s00270-018-1971-7)
Supplement: Supplementary file 1 — Supplementary material 1 (DOCX 15 kb) [file 270_2018_1971_MOESM1_ESM.docx]

**Online Resource 1**

**Article title:** Conductivity Rise during Irreversible Electroporation: True Permeabilization or Heat?

**Journal name:** CardioVascular and Interventional Radiology (CVIR)

**Author names & affiliations:** AH Ruarus ^1^, LGPH Vroomen ^1^, RS Puijk ^1^, HJ Scheffer ^1^, TJC Faes ^1^, MR Meijerink ^1^

^1^ Department of Radiology and Nuclear Medicine, VU University Medical Center, Amsterdam, The Netherlands

**E-mail address corresponding author:** a.ruarus@vumc.nl

**Hypotheses Materials and Methods**

*Cool-down period*

In an acellular medium, a current increase cannot be accountable to membrane permeabilization and therefore must be the exclusive result of the temperature increase. As a consequence, the current must return to its baseline value after a sufficient cool-down period in which temperature has returned to baseline. In contrast, in a cellular medium, the periprocedural current increase may be due to either permeabilization or temperature, and, as a result of the permeabilization effects, the current should ***not*** return to baseline values after a cool-down period. Comparing the current after the cool-down period with the periprocedural current thus provides insight in the contribution of the effects of permeabilization and temperature.

*Baseline temperature*

If temperature would not attribute to a periprocedural current increase, the observed current must ***not*** differ between the different baseline temperatures (20 and 40°C) of the tissue-mimicking gel phantom.
